# Pituitary response to thyrotropin releasing hormone in children with overweight and obesity

**DOI:** 10.1038/srep31032

**Published:** 2016-08-03

**Authors:** Jesse Rijks, Bas Penders, Elke Dorenbos, Saartje Straetemans, Willem-Jan Gerver, Anita Vreugdenhil

**Affiliations:** 1 Centre for Overweight Adolescent and Children’s Healthcare (COACH), Department of Paediatrics, Maastricht University Medical Centre, P. Debyelaan 25, 6229 HX Maastricht, The Netherlands; 2 School of Nutrition & Translational Research in Metabolism (NUTRIM), Maastricht University, Universiteitssingel 6229 HR, Maastricht, The Netherlands

## Abstract

Thyroid stimulating hormone (TSH) concentrations in the high normal range are common in children with overweight and obesity, and associated with increased cardiovascular disease risk. Prior studies aiming at unravelling the mechanisms underlying these high TSH concentrations mainly focused on factors promoting thyrotropin releasing hormone (TRH) production as a cause for high TSH concentrations. However, it is unknown whether TSH release of the pituitary in response to TRH is affected in children with overweight and obesity. Here we describe TSH release of the pituitary in response to exogenous TRH in 73 euthyroid children (39% males) with overweight or (morbid) obesity. Baseline TSH concentrations (0.9–5.5 mU/L) were not associated with BMI z score, whereas these concentrations were positively associated with TSH concentrations 20 minutes after TRH administration (r^2^ = 0.484, p < 0.001) and the TSH incremental area under the curve during the TRH stimulation test (r^2^ = 0.307, p < 0.001). These results suggest that pituitary TSH release in response to TRH stimulation might be an important factor contributing to high normal serum TSH concentrations, which is a regular finding in children with overweight and obesity. The clinical significance and the intermediate factors contributing to pituitary TSH release need to be elucidated in future studies.

In children with overweight and obesity thyroid stimulating hormone (TSH) concentrations are often higher compared to TSH concentrations of lean children^1^,^2^. Also, TSH concentrations above the normal range in combination with normal free thyroxin (fT4) concentrations are common in children with overweight and obesity^1^,^2^,^3^,^4^,^5^,^6^. Both TSH concentrations in the high normal range and TSH concentrations above the cut-off value for normal are associated with obesity related complications, including increased cardiovascular disease risk and non-alcoholic fatty liver disease^1^,^2^,^3^,^4^,^5^,^6^,^7^. Various theories have been postulated trying to explain the cause of the frequently found TSH concentrations in the high normal range and above the normal range, including leptin-mediated production of pro-thyrotropin releasing hormone (pro-TRH) and thyroid hormone resistance^6^,^8^,^9^. However, none of these hypotheses have been proven conclusively, and studies investigating the functioning of the hypothalamic-pituitary-thyroid (HPT) axis are limited in children with overweight and obesity. Interestingly, in adults with obesity an increased TSH release of the pituitary in response to exogenous thyrotropin releasing hormone (TRH) stimulation as compared to lean adults has been reported^10^,^11^,^12^. This suggests that HPT-axis functioning, and especially the pituitary functioning, might be altered in subjects with obesity. Possibly, pro-inflammatory cytokines affect the HPT-axis, which has also been suggested as the link between TSH concentrations and increased cardiovascular disease risk in subjects with obesity^13^,^14^. In children with overweight and obesity, studies investigating the pituitary TSH release in response to exogenous TRH stimulation are scarce and limited to small study populations^15^,^16^,^17^. In this study we evaluated the TSH release of the pituitary in response to exogenous TRH stimulation in a large group of children with overweight and obesity.

## Results

Seventy-three children (39% males) with overweight and obesity, and a mean age of 12.7 ± 3.1 years were enrolled. Baseline serum TSH concentrations were 2.7 (1.5–4.1) mU/L in the children with overweight, 3.4 (1.5–5.0) mU/L in the children with obesity, and 2.5 (0.9–5.5) mU/L in the children with morbid obesity. FT4 concentrations were within normal range in all children (13.3 ± 2.0 pmol/L). All participant characteristics are presented in Table 1.

**Figure 1:**
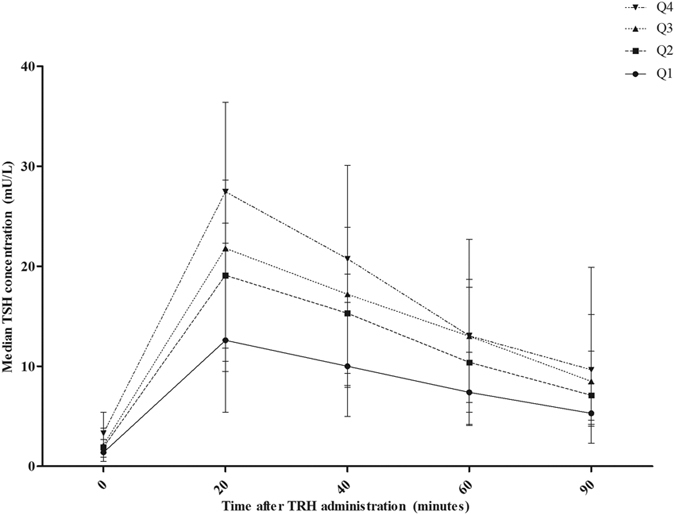
TSH release of the pituitary in response to exogenous TRH stratified into baseline serum TSH concentrations quartiles. Baseline serum TSH concentrations were stratified for quartiles: Q1 = < 2.05 mU/L (*n* = 18); Q2 = 2.05–2.99 mU/L (*n* = 17); Q3 = 3.00–3.69 mU/L (*n* = 19); Q4 = > 3.69 mU/L (*n* = 19). The TSH iAUC during the TRH test was significantly different between the baseline serum TSH concentration quartiles (*p* < 0.001). Post-hoc analysis showed a significant difference between quartile 1 and quartile 3 (*p* = 0.002), and between quartile 1 and quartile 4 (*p* < 0.001). Baseline serum TSH concentrations were within the normal range in all children based on age specific references ranges^31^. TSH = thyroid stimulating hormone; TRH = thyrotropin releasing hormone; Q1 = quartile 1; Q2 = quartile 2; Q3 = quartile 3; Q4 = quartile 4.

Baseline serum TSH concentrations were stratified into quartiles to evaluate TSH response of the pituitary in response to exogenous TRH stimulation for children in the higher and lower normal ranges of baseline serum TSH concentration (quartile 1 = < 2.05 mU/L; quartile 2 = 2.05–2.99 mU/L; quartile 3 = 3.00–3.69 mU/L; quartile 4 = > 3.69 mU/L). This is shown in Fig. 1. The TSH iAUC during the TRH test was significantly different between children in the different quartiles (p < 0.001). Post-hoc analysis showed a significant difference between quartile 1 and quartile 3 (p = 0.002), and between quartile 1 and quartile 4 (p < 0.001).

**Figure 2:**
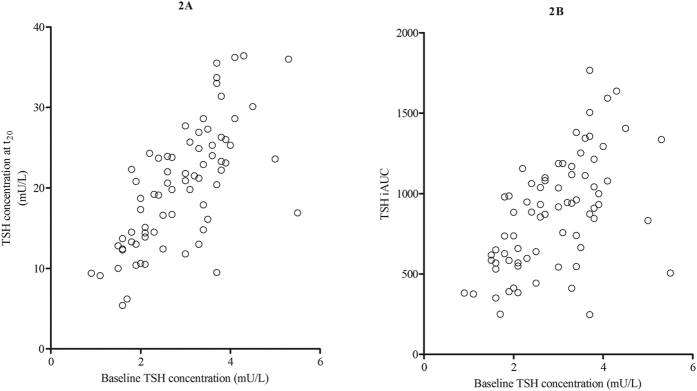
Baseline serum TSH concentrations in association with TSH concentrations at t_20_ and the TSH iAUC. (**A**) Association of baseline TSH concentrations and the TSH concentrations at t20 (*r*^2^ = 0.484, *p* < 0.001), *n* = 73. (**B**) Association of baseline TSH concentrations and the TSH iAUC during the TRH stimulation test (*r*^2^ = 0.307, *p* < 0.001), *n* = 73. Baseline serum TSH concentrations were within the normal range in all children based on age specific references ranges^31^. TSH = thyroid stimulating hormone; TRH = thyrotropin releasing hormone; *t*_20_ = 20 minutes after TRH administration; iAUC: incremental area under the curve.

Baseline serum TSH concentrations were positively associated with both, serum TSH concentrations twenty minutes after TRH administration (t_20_) (r^2^ = 0.484, p < 0.001) and the TSH incremental area under the curve (iAUC) during the TRH stimulation test (r^2^ = 0.307, p < 0.001) (Fig. 2A,B). Furthermore, the serum TSH concentration at t_20_ showed an inverse association with age (r^2^ = 0.056; p = 0.044), while no associations were found between age and the TSH iAUC during the TRH stimulation test. There were no gender differences regarding baseline serum TSH concentrations, serum TSH concentrations at t_20,_ and TSH iAUC during the TRH stimulation test.

BMI z-score and waist circumference z-score showed no significant associations with baseline serum TSH concentrations, serum TSH concentrations at t_20_, or the TSH iAUC during the TRH stimulation test. Significant inverse associations between serum c-reactive protein (CRP) concentrations and serum TSH concentrations at t_20_ (r^2^ = 0.142, p = 0.01), and the TSH iAUC during the TRH stimulation test (r^2^ = 0.124, p = 0.007) were demonstrated. Plasma interleukin 6 (IL-6) concentrations were also significantly negative associated with serum TSH concentrations at t_20_ (r^2^ = 0.118, p = 0.026, respectively) and with the TSH iAUC during the TRH stimulation test (r^2^ = 0.116, p = 0.009).

## DISCUSSION

**Figure 3:**
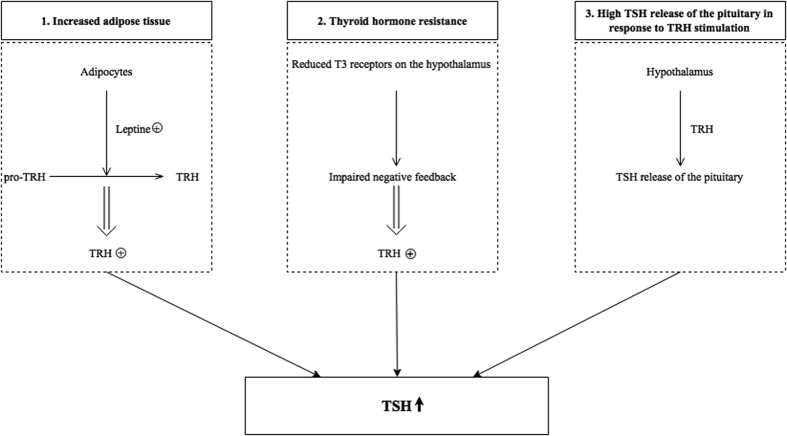
Postulated mechanisms contributing to TSH concentrations in children with overweight and obesity. TRH = thyrotropin releasing hormone; T3 = triiodothyronine; TSH = thyroid stimulating hormone.

This is the first study investigating pituitary TSH release in response to exogenous TRH stimulation in a large group of euthyroid children with overweight and obesity. A positive association between baseline serum TSH concentrations and TSH release of the pituitary in response to exogenous TRH stimulation was demonstrated. This suggests that TSH release of the pituitary in response to TRH stimulation might be an important factor contributing to the frequently found high normal baseline serum TSH concentrations in children with overweight and obesity (Fig. 3), which is associated with several obesity related complications^1^,^2^,^3^,^4^,^5^,^6^,^7^.

Studies investigating TSH release of the pituitary in response to exogenous TRH stimulation in children with overweight and obesity are limited to small study populations^15^,^16^,^17^. In line with our findings in children, studies in adults with obesity demonstrated a higher TSH release in response to exogenous TRH stimulation as compared to lean adults^10^,^11^,^12^. Besides these HPT axis alterations, hyperactivity of the hypothalamic-pituitary-adrenal (HPA) axis has been described in adults with obesity when adrenocorticotropic hormone (ACTH) and cortisol concentrations were studied^18^,^19^,^20^. Since previous studies have shown that the HPA-axis can be influenced by pro-inflammatory cytokines^21^,^22^ and the fact that obesity is characterized by a chronic state of low-grade inflammation^23^, it is tempting to suggest that presence of pro-inflammatory mediators might also play a role in the alterations in the other hypothalamic axes. However, results of this study showed that serum CRP concentrations and plasma IL-6 concentrations were negatively associated with serum TSH concentrations at t_20_ and with the TSH iAUC during the TRH stimulation test, ruling out inflammatory stimulation as a contributing factor to high pituitary TSH release. Interestingly, this study showed that TSH release of the pituitary in response to exogenous TRH stimulation and high normal baseline serum TSH concentrations are not simply the consequence of excess body weight, since BMI z-score and waist circumference z-score were not associated with baseline serum TSH concentrations, serum TSH concentrations at t_20_, or the TSH iAUC during the TRH stimulation test. This reinforces the findings of Aeberli *et al*. who demonstrated no associations between baseline TSH concentrations and the amount of excess body weight or fat in children with obesity^3^.

Thus, the results of the current study showed that neither pro-inflammatory cytokines nor the amount of excess body weight is associated with high TSH release of the pituitary in response to exogenous TRH stimulation. Considering the evidence that in mice leptin contributes to regulation of HPT-axis activity^24^, there might also be a role for adipokines influencing the HPT-axis and pituitary TSH response to TRH in humans. Since the objective of this study was to evaluate the direct effect of TRH on pituitary TSH release, adipokines concentrations were not determined and cannot be evaluated as intermediate factors to high pituitary TSH release in this study. Furthermore, a recent review suggested that the HPT-axis activity is influenced by nutritional status and stressful situations including physical activity^25^. Oppert *et al*. also demonstrated an increased pituitary TSH release in response to exogenous TRH stimulation in young adults during long-term overfeeding as compared to the preoverfeeding TSH release^26^. Feeding status was not assessed in our study, but it is tempting to suggest that children with overweight and obesity are often exposed to overfeeding. Future studies are necessary to determine which factors might also affect pituitary TSH release in children with overweight and obesity.

In conclusion, baseline serum TSH concentrations are associated with TSH release of the pituitary in response to exogenous TRH stimulation in euthyroid children with overweight and obesity. The clinical significance and the intermediate factors contributing to pituitary TSH release need to be elucidated in future studies.

## Methods

### Study participants

This cross-sectional study was designed and conducted within the setting of the Centre for Overweight Adolescent and Children’s Healthcare (COACH) at the Maastricht University Medical Centre (Maastricht UMC+). Within COACH, the health status of children with overweight, obesity, and morbid obesity is evaluated, and they are monitored and guided as described previously^27^. All children received a TRH stimulation test at the beginning of their participation in the COACH program. Children without a complete TRH stimulation test were excluded in this retrospective study. Further, children with baseline serum TSH concentrations above the normal range and children with thyroid diseases were excluded. Finally, 73 children were eligible for inclusion. Disease-related causes for overweight were ruled out in all children. The study was conducted in concordance with the guidelines laid down in the Declaration of Helsinki and approved by the medical ethical committee of the Maastricht UMC+. Informed consent was obtained from all subjects or their parent or legal guardian.

### Participant characteristics

Anthropometric data were obtained while children were barefoot and wearing only underwear. Body weight was determined using a digital scale (Seca) and body length was measured using a digital stadiometer. BMI was calculated and BMI z-scores were obtained using a growth analyser (Growth Analyser VE). Based on the International Obesity Task Force criteria children were classified as overweight, obese, or morbidly obese^28^. Waist circumference was measured with a non-elastic tape at the end of a natural breath at midpoint between the top of the iliac crest and the lower margin of the last palpable rib. Hip circumference was measured at the widest portion of the buttocks. Waist- and hip circumference z-scores were determined^29^, waist-to-hip (WHR) ratio was calculated, and ethnicity was defined^30^. Both during history taking and physical examination, there were no indications for the presence of an incurrent infection in all children.

### Thyroid function

Venous blood samples were collected after a minimum of 8 hours overnight fasting for the determination of baseline serum TSH and fT4 concentrations. Serum TSH concentrations were determined with the Cobas 8000 modular analyser (Roche), and serum fT4 concentrations were determined with the Autodelfia fluoroimmunoassay system (PerkinElmer). Serum TSH concentrations were considered within the normal range or above the normal range based on age specific references ranges^31^. Serum fT4 concentrations were considered normal between the range of 8–18 pmol/L.

### TRH stimulation test

At the start of the TRH stimulation test non-fasting serum TSH concentrations (t_0_) were determined. A bolus of 200 μg TRH was given intravenously, subsequently venous blood samples were obtained to determine serum TSH concentrations at 20 minutes (t_20_), 40 minutes (t_40_), 60 minutes (t_60_), and 90 minutes (t_90_) after the TRH administration.

### Inflammatory markers

CRP concentrations were determined with the Cobas 8000 modular analyser (Roche). Plasma pro-inflammatory cytokines monocyte protein 1 (MCP-1), IL-6, and interleukin 8 (IL-8), were measured with a commercially available Multi Spot ELISA assay (Meso Scale Discovery).

### Statistical analysis

All statistical analyses were performed using SPSS 23.0 for Windows (SPSS Inc). Shapiro-Wilk test was performed to test for normality. Serum baseline TSH concentrations were stratified into quartiles. The TSH iAUC was calculated using the trapezoidal method. A one-way analysis of variance (ANOVA) with Bonferroni as post hoc analysis was used to evaluate differences in iAUC between serum baseline TSH concentration quartiles. Associations between variables were determined by linear regressions models. Since TSH concentrations are age dependent^31^ associations were adjusted for age. A *p*-value below 0.05 was considered statistically significant. Data are presented as mean with standard deviation or as median with the minimum and maximum.

### Clinical trial registration

Clinical trial registration at ClinicalTrial.gov; Registration Number: NCT02091544.

## Additional Information

**How to cite this article**: Rijks, J. *et al*. Pituitary response to thyrotropin releasing hormone in children with overweight and obesity. *Sci. Rep.* **6**, 31032; doi: 10.1038/srep31032 (2016).

## Figures and Tables

**Table 1:** Characteristics of the study participants.

*Age*	12.7 ± 3.1
*Male/Female, %*	39/61
*Caucasian*^a^*, %*	76
*BMI z-score*	3.51 ± 0.74
*Overweight/obese/morbidly obese*^b^, *%*	17/38/45
*Waist circumference z-score*	6.8 ± 2.6
*Hip circumference z-score*	4.6 ± 1.9
*Waist-to-hip ratio*	0.95 ± 0.1
*Baseline TSH concentrations, mU/L*	3.0 (0.9–5.5)
*FT4, pmol/L*	13.4 ± 2.0
*TSH concentrations t*_*0*_*, mU/L*	2.0 (0.5–5.4)
*TSH concentrations t*_*20*_*, mU/L*	20.5 (5.4–36.4)
*TSH concentrations t*_*40*_*, mU/L*	15.4 (5.0–30.1)
*TSH concentrations t*_*60*_*, mU/L*	11.0 (4.1–22.7)
*TSH concentrations t*_*90*_*, mU/L*	7.7 (2.3–19.9)
*TSH iAUC during TRH stimulation test*	874 ± 351
*C-reactive protein, mg/L*	4.0 (1.0–51.0)
*MCP-1, pg/mL*	132.7 (66.7–372.6)
*IL-6, pg/mL*	1.09 (0.23–2.51)
*IL-8, pg/mL*	3.01 (1.14–282.81)

Data presented as mean ± SD or as median (minimum-maximum); n = 73. Baseline serum TSH concentrations were within the normal range in all children based on age specific references ranges^31^. TSH = thyroid stimulation hormone; TRH = thyrotropin releasing hormone; fT4 = free thyroxin; t_x_ = x minutes after TRH administration; iAUC = incremental area under the curve; MCP-1 = monocyte protein-1; IL-6 = interleukin 6; IL-8 = interleukin 8.

^a^According to the Dutch Central Agency for Statistics^30^.

^b^According to the International Obesity Taskforce Criteria^28^.
